# New Insights into the Role of Tyro3, Axl, and Mer Receptors in Rheumatoid Arthritis

**DOI:** 10.1155/2020/1614627

**Published:** 2020-01-19

**Authors:** Sara Pagani, Mattia Bellan, Daniele Mauro, Luigi Mario Castello, Gian Carlo Avanzi, Myles J. Lewis, Pier Paolo Sainaghi, Costantino Pitzalis, Alessandra Nerviani

**Affiliations:** ^1^Centre for Experimental Medicine & Rheumatology, William Harvey Research Institute, Barts and The London School of Medicine and Dentistry, Queen Mary University of London, London, UK; ^2^Department of Translational Medicine, Università del Piemonte Orientale (UPO), Novara, Italy; ^3^Internal Medicine Division, Immunorheumatology Unit, CAAD (Center for Autoimmune and Allergic Diseases), “Maggiore della Carità” Hospital, Novara, Italy; ^4^IRCAD (Interdisciplinary Research Center of Autoimmune Diseases), Novara, Italy

## Abstract

Rheumatoid Arthritis (RA) is the most common chronic inflammatory autoimmune disease involving joints. Among several pathogenic mechanisms, the impairment of homeostatic regulators of inflammation seems to be critically important to sustain persistent infiltration and activation of immune and stromal cells within the diseased synovium. Tyrosine kinase receptors Tyro3, Axl, and Mer are members of the TAM family. Upon binding their ligands Growth Arrest-Specific gene 6 (Gas6) and Protein S (ProS1), TAM receptors (TAMs) exert numerous and diverse biologic functions. Activated Axl and Mer, for instance, can negatively regulate the inflammatory cascade and mediate phagocytosis of apoptotic cells, contributing to prevent the development of autoimmunity. Thus, a role for TAMs has been hypothesized in RA. In this review, we will summarise unmet clinical needs in RA, depict the biology of TAMs and TAM ligands, focussing on their ability to regulate the immune system and inflammation cascade, and finally offer an overview of the state-of-the-art literature about the putative role of TAM axis in RA.

## 1. Introduction

Rheumatoid Arthritis (RA) is a chronic inflammatory autoimmune disease characterised by persistent inflammation of diarthrodial joints [1]. Despite significant advances in the understanding and management of RA, further studies evaluating novel pathogenic pathways and therapeutic targets are needed to improve the clinical outcome of patients. Among several mechanisms, impairment of homeostatic regulators of inflammation seems to be critically important to sustain the persistent cellular infiltration and activation of immune and stromal cells within the diseased synovium [2]. Tyro3, Axl, and Mer are three tyrosine kinase receptor (TKR) members of the TAM family, which can be activated by binding their cognate ligands Growth Arrest-Specific gene 6 (Gas6) and Protein S (ProS1) [3]. TAM receptors (TAMs) have been implicated in several biological processes such as inhibition of apoptosis and promotion of cell survival and proliferation [4, 5], inhibition of granulocytes adhesion to the endothelium [6], and stabilisation of blood clots [7]. Furthermore, and of particular importance in the context of RA, TAMs can also finely regulate the inflammatory cascade [8] and mediate the engulfment of apoptotic corpses [9], contributing to prevent the development of autoimmune reactions.

Here, we will initially summarise unmet clinical needs in RA (Section 2) and describe the biology of TAMs and TAM ligands (Section 3). We will then focus on TAMs' ability to control the immune system and inhibit the inflammatory cascade (Section 4). Finally, we will offer an overview of the state-of-the-art literature about the putative role of the TAM axis in RA (Section 5).

## 2. Unmet Needs in Rheumatoid Arthritis

RA is the most common chronic inflammatory autoimmune disease affecting joints. If not adequately treated, RA eventually causes long-term disabilities and poor quality of life [1]. RA pathogenesis is multifactorial and only partially understood. In the prearticular phase of the disease, characterised by systemic loss of the immune tolerance, autoantibodies directed against arthritogenic peptides are generated in genetically susceptible subjects [10]. Subsequently, multiple factors such as viral infections, microvascular defects, and local microtraumas likely contribute to shifting the pathogenic process from the periphery to the joints, hence initiating the articular phase of the disease [2].

Within the affected joint, autoantibodies bind their cognate antigens and activate the complement cascade, ultimately triggering proinflammatory reactions mediated by resident synovial cells and immune cells recruited from peripheral blood. This persistent infiltration of the synovial membrane by inflammatory cells is, at least partially, self-sustained by intrinsic and/or acquired defects of homeostatic regulatory mechanisms operating a negative feedback on the inflammatory cascade [2, 11].

Over the last two decades, thanks to the introduction of biologic agents into the therapeutic scenario, the clinical outcome of RA patients has critically improved. Nevertheless, substantial unmet clinical needs remain to be addressed for further refining the diagnosis and ameliorating the prognosis of patients. For instance, biomarkers able to accurately predict the diagnosis, severity, and progression of RA have yet to be defined. Moreover, a still significant percentage of patients, despite being aggressively treated with multiple agents, fail to reach a low-disease activity or remission status [12]. In the era of precision medicine, the identification of predictors able to guide the choice of the best drug for the right patient represents one of the most important goals of ongoing trials. Even if exciting news is currently coming from the analysis of the cellular and molecular content of the diseased synovial tissue [13], further investigations are still required. To date, a few studies have explored TAMs' pathogenic role and potential diagnostic and prognostic value in RA. As described below, the biological features of TAMs and TAM ligands make this system a promising candidate biomarker and a future therapeutic target in RA.

## 3. Biology of TAM Receptors and Ligands

### 3.1. Structure, Expression, and Activation of TAM Receptors and Ligands

The acronym TAM is derived from the names of the three RTK members of the family: Tyro3, Axl, and Mer [14]. Structurally, all TAMs are considerably similar and contain the following: an extracellular amino-terminal region carrying tandem immunoglobulin-related domains, which mediate ligands' binding, followed by two fibronectin type III repeats; a single-pass transmembrane domain; and a catalytically competent tyrosine kinase intracellular domain [15, 16]. TAMs had been considered “orphan” receptors until 1995 when their ligands ProS1 and Gas6 were identified [17]. Gas6 can bind and activate all three TAMs, however, with different degrees of affinity (Axl>Tyro3>>Mer); conversely, ProS1 is the preferential ligand for Tyro3 and Mer but has a significantly lower affinity for Axl [17, 18].

Although Axl, Mer, and Tyro3 mRNA can be detected in embryonic tissues [19], TAMs are dispensable for embryonic growth and nonessential for the viability of the foetus as demonstrated by the healthy birth of triple TAM knockout (KO) mice [20]. In adult tissues, TAMs are broadly expressed but can be primarily found in the nervous and reproductive systems, retinal cells, and hematopoietic lineages [21]. Myeloid cells (i.e., monocytes/macrophages and dendritic cells (DCs)), in particular, display TAMs on their surface [8, 22] though with distinctive features. On the one hand, Axl and Tyro3 are usually upregulated by monocyte-derived DCs [23] and, among them, Axl is preferentially induced by GM-CSF and IFN-*α* stimulation [24]. On the other hand, Mer is a typical macrophage receptor predominantly expressed by anti-inflammatory macrophage M2c, obtained *in vitro* by treating monocytes with M-CSF and IL-10 [25]. Overall, both Axl and Mer seem to be gradually acquired as monocytes differentiate into DCs and macrophages, respectively. Interestingly, despite being expressed by several neoplastic lymphocytes, TAMs are almost undetectable in nonpathologic B and T cells [21], except for specific subsets of B cells [26] and CD4+CD25+ regulatory T cells [27].

Depending on which cells or tissues are expressed by, TAMs can activate different intracellular pathways and mediate a wide range of biological functions [28]. In most nonsentinel cells, activation of TAM tyrosine kinases is coupled to the downstream activation of the phosphoinositide-3-kinase (PI3K)/AKT pathway. Conversely, in antigen-presenting cells (APCs) and other immune cells harbouring the Type I Interferon Receptor (IFNAR), the JAK/STAT signalling becomes the preferential downstream pathway [29]. As mentioned above, TAMs are activated upon binding their cognate ligands Gas6 and ProS1. However, besides this conventional ligand-dependent stimulation, in nonphysiological circumstances of overexpression, Axl activation can also occur without binding its ligand but through the aggregation of extracellular domains and subsequent reciprocal autophosphorylation [30].

### 3.2. Regulation of TAM Receptors: Shedding Mechanisms and Epigenetic Modulation

Several mechanisms can critically regulate TAM protein expression, including cleavage of extracellular domains and epigenetic control of mRNA translation. Concerning the former, two A disintegrin and metalloproteinases (ADAM), namely ADAM10 and 17, are the principal enzymes involved in the generation of soluble Axl (sAxl) and soluble Mer (sMer) extracellular domains [31, 32]. TAMs shedding may have important physiological and pathological implications: in fact, because of Axl high affinity for its ligand Gas6, sAxl behaves as a potent decoy receptor for circulating Gas6. Hence, in the presence of excessive cleavage, not only the amount of functional transmembrane receptors is reduced but also the availability of Gas6 is impaired as it is sequestered by sAxl [33]. Interestingly, proinflammatory stimuli such as phorbol 12-myristate 13-acetate (PMA) [31] and lipopolysaccharide (LPS) [32] are inducers of Axl and Mer shedding, respectively. In this context, the highly inflamed articular microenvironment during RA might play an essential role by enhancing TAM cleavage and, eventually, altering their homeostatic regulation. Furthermore, it has been shown that rheumatoid synovium expresses higher levels of both ADAM-10 and ADAM-17 compared with osteoarthritic and healthy joints [34]. Besides, RA-derived synovial fibroblasts further upregulate ADAMs' expression upon stimulation with proinflammatory cytokines in comparison with resting cells [35]. Shedding of the ectodomain can unmask secondary cleavage sites that, if activated, release soluble intracellular domains; recently, it has been suggested that all three TAMs have intramembrane cleavage sites potentially targeted by gamma-secretase shedding complexes [36].

Importantly, since soluble TAM ectodomains can be easily quantified, they may become valuable diagnostic and/or prognostic biomarkers in the context of inflammatory and autoimmune conditions. Indeed, significant variations of plasmatic levels of TAMs and ligands have been described in numerous pathological conditions. For instance, raised concentrations of soluble TAMs associate with lupus [37–39], Sjogren's syndrome [40, 41], and RA [42, 43]; Gas6 is heightened in a multitude of diseases, such as inflammatory autoimmune demyelinating diseases [44], Alzheimer's disease [45], and hepatic fibrosis [46]. Higher levels of Gas6 also predict oesophageal varices in patients affected by hepatitis C virus (HCV) liver disease [47] and correlate with disease severity in multiple sclerosis [48] and renal involvement in systemic lupus erythematosus (SLE) [49]. Conversely, other authors have found lower Gas6 plasmatic concentrations in lupus [50], Behcet's disease [51], and inflammatory bowel diseases [52] in comparison with healthy controls. Heterogeneity of the cohorts included in the studies might account for these discrepancies since different ethnicity, stage of the disease, previous treatments, and comorbidities can influence the level of expression of both soluble TAMs and TAM ligands.

Epigenetic control, which is acquiring increasing importance, is another mechanism able to regulate TAM protein expression. Briefly, miRs are small noncoding RNA that can modulate the mRNA translation of target genes, hence altering their effector pathways. Research of Axl-modulating miRs was initially performed in malignant cells and tissues and provided a fascinating list of candidates [53]: among them, miR-34a has been selected and studied also in the context of inflammation. Interestingly, it was found that the inhibition of miR-34a in macrophages caused the downregulation of proinflammatory cytokines' release [54] and, in line with these results, that RA DCs were characterised by unrestrained activation of miR-34a driving the uncontrolled production of inflammatory molecules secondary to Axl repression [55].

## 4. TAM Receptors as Regulators of the Immune System

TAMs' ability to maintain immune system homeostasis and control inflammatory responses in adult tissues was firstly suggested by the phenotype of Mer kinase-dead (MerKD) mice, characterised by an excessive production of Tumor Necrosis Factor (TNF) *α* upon LPS stimulation and death by endotoxic shock caused by less-lethal doses of LPS [56]. Later on, it was also shown that mutants lacking all three TAMs (known as TAM^−/−^ mice) developed multiorgan signs and symptoms typical of autoimmune inflammatory diseases [20, 21]. TAM^−/−^ mice became progressively blind and sterile and showed gradual enlargement of secondary lymphoid organs caused by an uncontrolled proliferation of B/T lymphocytes [21]; at about six months of age, they displayed a wide range of full-blown clinical, serological, and histological manifestations including immunoglobulin deposits in glomeruli, circulating autoantibodies, vasculitic skin lesions, alopecia, and swollen joints [20, 21].

In the attempt to explain these broad pathological manifestations, two essential TAM-regulated functions were identified and described: the inhibition of Toll-Like-Receptors (TLRs) induced inflammatory cascade and the uptake of apoptotic cells by APCs. The impairment of these mechanisms in the absence of TAMs could, at least partially, recapitulate and explain TAM^−/−^ phenotype.

### 4.1. Inhibition of Toll-Like Receptor- (TLR-) Mediated Inflammation

Upon being bound by their ligands, TLRs respond by enhancing the release of proinflammatory cytokines, which are crucial for host defence mechanisms against microbial pathogens. On the other hand, failure of TLR fine-tuning causing their unrestrained activation may generate an inflamed environment promoting autoimmunity [57]. APCs like DCs and macrophages use TAMs to regulate and switch off inflammatory reactions secondary to TLR stimulation, thus preventing the chronic activation of TAM-expressing cells [22].

Molecular mechanisms by which TAMs exert this inhibitory function have been particularly well studied in DCs expressing Axl. The initial inflammatory rush provoked by TLR activation and typically exploiting the IFNAR/STAT1 as downstream activator signal can, in turn, also prompt Axl upregulation. Once Axl has been exposed on the cell membrane and activated by its ligand, it can complex with the IFNAR and usurp the IFNAR/STAT1 machinery from TLRs, eventually determining the switch from a pro- to an anti-inflammatory phenotype of the cell. Coupling of Axl with IFNAR upregulates the transcription of inhibitory factors, for instance, the suppressors of cytokine signalling family 1/3 (SOCS1/3) [22]. Mer is likewise important for the inhibition of inflammation in macrophages [58] and macrophage-like cell lines [8]. As reported by Alciato et al., Mer activation by its ligand Gas6 drives the downregulation of LPS-induced production of TNF-*α* and IL-6 in monocyte-derived macrophages and U937-derived macrophage-like cells by triggering PI3K/AKT and NF-kappa B pathways [8]. Furthermore, as suggested by Zizzo et al., the Mer/Gas6 axis not only can prevent proinflammatory cytokines' release but also induce the expression of anti-inflammatory mediators (i.e., IL-10) by M2c anti-inflammatory macrophages. Ultimately, Mer/Gas6-induced IL-10 represents a positive feedback loop for M2c cell homeostasis, and it is critical for maintaining an anti-inflammatory and immune-tolerant environment [25]. TAMs' ability to contain the overproduction of TNF*α* and IL-6 is particularly important in the context of RA since both of these cytokines are abundantly produced within the rheumatoid synovial tissue and sustain the chronic inflammatory process [59, 60]. Clinical efficacy of biologic agents targeting TNF*α* and IL-6 (e.g., infliximab [61] and tocilizumab [62], respectively) further confirms the detrimental effects played by these molecules in RA.

### 4.2. Phagocytosis of Apoptotic Cells

The second TAM-mediated mechanism relevant to the immune system regulation is the phagocytosis of apoptotic cells, also called efferocytosis. Removal of apoptotic debris is crucial for maintaining adult tissues healthy and functional. In mice lacking TAMs, initial evidence of defective efferocytosis can be observed in tissues and organs characterised by high cellular turnover, for instance, retina, reproductive, and immune system. Failure to phagocyte apoptotic residues in these tissues clinically manifests with blindness, sterility, and pathological enlargement of secondary lymphoid organs, respectively [20]. Unremoved apoptotic cells are a source of autoantigens and can drive the development of autoimmunity [63, 64], thus underpinning a strong link between the absence of TAMs and the broad-spectrum autoimmune manifestations observed in the triple KO.

The mechanism of efferocytosis used by TAMs is peculiar and carefully regulated. During apoptosis, dying cells expose phosphatidylserine (PtDSer) on their membrane as an “eat me” signal, which makes phagocytes able to discriminate them from other necrotic or healthy cells. TAM ligands Gas6 and Pros1 allow TAM-mediated efferocytosis by binding the PtDSer residues on apoptotic cells via their Gla domains and TAMs on APCs via their amino-terminal region. In this way, TAM ligands function as a “bridge” between apoptotic cells and TAM-expressing phagocytes [65]. Mer was the first TAM receptor discovered to mediate efferocytosis thanks to early experiments performed using MerKD mice. MerKD-derived macrophages were indeed unable to adequately clear thymocytes, but fascinatingly, their phagocytosis deficiency was restricted to apoptotic cells and independent of Fc receptor. Altogether, these findings suggested a critical and exclusive role of Mer in the clearance of apoptotic bodies [66].

Even though Mer has been historically considered the only TAM responsible for the efferocytosis process, recent data highlighted that, under certain circumstances, also other members of the TAM family can acquire phagocytic activity [33]. Depending on the surrounding microenvironment, the same cell type can upregulate either Mer or Axl: in the presence of tolerogenic or immunosuppressive stimuli, Mer is the principal mediator of efferocytosis, and its final aim is maintaining normal tissue cellularity in physiological conditions or upon anti-inflammatory treatment; conversely, following proinflammatory activation of phagocytes, Mer is downregulated and, in turn, Axl takes control of the process [33]. Notably, in RA synovial tissue, NF-*κ*B is strongly activated and provides a robust prosurvival signal and sustains the resistance to apoptosis [67]. Thus, once again, a strong link between one of TAM-mediated functions and the development of RA exists, suggesting that TAMs may be involved in the pathogenesis of the disease.

### 4.3. TAM Receptors Link the Innate and Adaptive Immunity

Once activated, cells of the adaptive immune system should feedback to innate immune cells to avoid their chronic and uncontrolled activation. Due to their characteristics, including the relatively late appearance in evolution, TAMs seem designated to represent this important connection.

In favour of this hypothesis, it has been recently showed that TAM ligand ProS1 is upregulated exclusively by activated (not resting) T cells and can inhibit their proliferation [68]. The mechanism proposed for explaining this process involves ProS1 ability to create a bridge between PtDSer, exposed by T cells only transitorily after being activated, and TAMs expressed by APCs [69]. ProS1, by binding PtDSer on T cells with its Gla domain and TAMs harboured by DCs with its SHBG domain, favours the connection between these two cell types from the adaptive and innate immune systems. The interaction between TAM/PtDSer drives an inhibitory signal that restrains the proinflammatory activation of DCs, hence limiting the production of cytokines such as IL-6 and TNF*α*, and will also ultimately inhibit T cells. As a proof of concept, preventing ProS1 to bind activated T cells triggered a rapid increase of activated DCs and proinflammatory molecule release [68].

Virtually, all TAM activities listed so far occur because of their expression by innate immune cells (either monocytes/macrophages or DCs). However, as an exception, a new TAM function involving CD4+CD25+ T regulatory (T-reg) cells has recently been described. T-reg cells exert their regulatory role largely by preventing the immune cell-induced organ damage. On the one hand, by suppressing autoreactive lymphocytes, T-reg cells are fundamental to avoid autoimmunity; on the other hand, however, an excessive activation of T-reg cells would lead to unhealthy immunosuppression. Defective expression, functionality, and generation of T-reg cells have been described in several autoimmune conditions including RA, in which they are highly present within the inflamed synovial tissue but reduced in the periphery [70]. Surprisingly, Axl and Mer have been detected on the surface of T-reg cells; once activated, Axl/Gas6 enhances the suppressive capacity of T-reg, supporting, once again, Gas6 anti-inflammatory abilities [27].

Overall, the interaction between TAMs, Gas6/ProS1 and innate/adaptive cells is a complex and finely-tuned process. Small changes to this delicate balance could favour the development of autoimmunity and chronic inflammation. Little is known at this regard in RA, but compelling evidence is growing, and future studies will hopefully further elucidate these critical aspects.

## 5. TAM Receptors Implications in Rheumatoid Arthritis

As mentioned above, the relevance of TAMs in the development and progression of inflammatory arthritis was initially hinted by the phenotype of TAM^−/−^ mice, characterised by broad-spectrum autoimmune manifestations, predominantly resembling SLE but also including inflammatory arthritis [21]. So far, human studies mainly focussed on TAMs' role in SLE showing that impairment in this receptor system is associated with lupus development, and soluble TAMs/ligands may be valuable diagnostic and/or prognostic biomarkers in this condition [3, 71]. Additional and new evidence about TAMs in RA has recently become available and is continuously growing. Over the last decades, several studies have investigated different models of arthritis in TAM single, double, and triple KO mice. One of the most accredited hypotheses that researchers are trying to prove implicates that dysregulation of the TAM axis triggers autoimmune reactions and the development of chronic inflammation within the synovial tissue. If this is correct, adjustments of the “aberrant” TAM system could represent a promising therapeutic target in arthritis.

Following the initial report of the triple TAM KO phenotype, a recent work on the same mice quite surprisingly showed that, in comparison with wild types (WT), KO littermates had neither macroscopic nor histological evidence of inflammatory arthritis in ankle joints until the age of 52 weeks [72]. As suggested by the authors, a different phenotype observed in a genotypically identical animal model may be justified by changes in the interplay between genetic and environmental factors, including, for example, improved cleanliness of facilities and modifications of the microbiota. The latter, in particular, could represent an exciting link with RA as the dysbiosis seems to be a promoter of inflammatory arthritis [73]. Despite not showing clinically evident arthritis, however, both adolescent and adult TAM^−/−^ mice had significantly more marked bone marrow oedema, which is an early sign of arthritis [72].

Further studies from the same group also showed that in a KRN serum transfer model of arthritis, the absence of Axl (Axl^−/−^) or Mer (Mer^−/−^) caused more severe disease in comparison with WT [74, 75]. Of note, the exacerbated pathology was observed only in ankles of Axl^−/−^ mice, whereas no effect was seen in the knees of Axl KO mice [75]. The histological analysis of the synovial tissue enabled a potential interpretation for this clinical outcome. While ankle synovium was characterised by high expression of Axl and a predominance of anti-inflammatory M2 macrophages, synovial tissue sampled from the knees had scant M2 macrophages and virtually absent Axl. Mer-deficient mice had instead aggravated disease in all the joints assessed [75].

The first *in vivo* evidence that TAMs might be therapeutically exploited to improve arthritis was provided in CIA mice treated with adenoviruses overexpressing ProS1 or Gas6. Intra-articular delivery of both TAM ligands Gas6 and ProS1 caused clinical and histological improvement by decreasing the production and release of Th1- and Th17-related proinflammatory cytokines (e.g., IL12/IFN*γ* and IL-23/IL-17, respectively) [76]. In contrast, only ProS1-overexpressing virus administered via a systemic route was able to improve the disease and reduce the number of splenic Th1-cells, leaving Th17 levels unaffected [76]. TAM ligands' effects may, therefore, depend on the delivery route and be “broader” when given locally. In line with these findings, it has been reported that the cytokine profile of *in vitro* stimulated peripheral blood CD4+ T cells isolated from Axl^−/−^/Mer^−/−^ mice is characterised by higher IFN*γ* but normal IL-17 [77].

The protective role played by Mer activation by its ligand ProS1 has been lately further confirmed in a KRN serum transfer arthritis model [74] and in a three-dimensional model of human synovium [74], hence enhancing the translational value of this discovery. On the other hand, Mer agonist antibodies were shown to have instead a detrimental effect on arthritis, which can be explained by their capacity of inhibiting Mer-mediated efferocytosis, proving that apoptotic cells removal is fundamental for homeostasis of the synovial tissue.

More recently, Culemann et al. found that Axl is expressed by a distinct subset of CX3CR1+ tissue-resident macrophages forming an immunological barrier at the synovial lining. These peculiar macrophages do not derive from circulating monocytes, proliferate locally, and share features with epithelial cells. By creating tight junctions and expressing anti-inflammatory receptors, these lining-layer macrophages tend to isolate the synovium and prevent the infiltration of inflammatory cells [78].

In contrast with the protective role hypothesised for Axl and Mer, induction of arthritis in Tyro3^−/−^ mice revealed that the third member of TAMs might instead play a proarthritic role. In particular, Tyro3 KO mice had less marked synovial fibroblast proliferation and osteoclast activation and were protected from bone damage in comparison with WT controls [79]. Furthermore, circulating levels of soluble Tyro3 positively correlated with disease activity and erosive burden in patients with RA [80]. It seems, therefore, that activated Tyro3 may be responsible for stimulating synovial hypertrophy, cartilage destruction, and bone erosion, suggesting a dual antithetic role for the TAM axis in arthritis depending on which receptor is activated, i.e., an anti-inflammatory effect in case of Axl or Mer but proerosive if Tyro3 is triggered. Of course, these observations should be taken into account when hypothesising a therapeutic exploitation of TAMs in inflammatory arthritis.

In contrast with a rather high number of studies in animal models, investigations of the TAM system in patients with RA have only recently returned a hot topic of research after an opening report published in 1999 when O'Donnel et al. found that Axl was expressed by a discrete subset of synoviocytes and vascular smooth muscle cells [43]. Our preliminary unpublished data have confirmed that Axl seems preferentially expressed by a subset of synovial lining macrophages, suggesting that it might play a similar “barrier” role as described in animal models of experimental arthritis.

It has been hypothesized that impaired TAM functioning prevents synovial cells to properly switch the inflammatory reactions off, thus triggering the development of chronic arthritis. The assumption of a defective expression of Axl in patients with RA was elegantly demonstrated in 2017 by Kurowska-Stolarska et al., who showed that CD1c+ DCs isolated from patients with RA have constitutively high levels of miR-34a and, subsequently, inhibited Axl expression in comparison with healthy donors [55]. Importantly, by inhibiting miR-34a, mice become resistant to arthritis, and DCs acquire back the ability to limit proinflammatory cytokine production.

As mentioned above, the Mer/Gas6 axis mediates anti-inflammatory effects in CD206+ CD163+ M2c macrophages by reducing the release of proinflammatory molecules like TNF or IL-6 [8] and, at the same time, by inducing anti-inflammatory mediators such as IL-10, which, in turn, can also positively regulate Gas6 continued secretion [25]. Interestingly, monocyte-derived macrophages isolated from RA patients treated with TNF-inhibitors showed downregulation of surface markers typically associated with inflammation (e.g., CD40 and CD80) but also upregulation of Mer, hence suggesting that, upon treatment, cells acquire the same anti-inflammatory properties as other Mer-positive macrophages. In line with this, *in vitro* studies confirmed that anti-TNF agents were able to inhibit proinflammatory cytokines and upregulate IL-10, activating a positive feedback mechanism involving the Gas6/Mer axis that, ultimately, limited the inflammatory cascade [81].

Recently, single-cell transcriptomic profiling of synovial tissue allowed the identification of several distinct subsets of synovial macrophages, differently expressed based on the nature and stage of the disease. In keeping with its postulated anti-inflammatory role, Mer was significantly highly expressed in osteoarthritic tissue compared to RA; moreover, among RA-specific macrophage subsets, Mer was upregulated in the so-called “anti-inflammatory” group [82]. Not surprisingly, therefore, emerging data suggest that synovial macrophages isolated from RA patients in remission are characterised by a CD163/CD206/Mer-positive signature [83].

The critical regulatory role played by TAM shedding and soluble TAM generation has gathered growing evidence. As mentioned above, indeed, quantification of circulating soluble TAMs and TAM ligands may represent a novel interesting biomarker system. For instance, in RA, sTyro3 serum levels were found elevated compared to healthy controls and correlated with rheumatoid factor titre, the number of swollen joints, and joint erosion scores [80]. The role and interpretation of sMer plasma levels, instead, are still controversial. In one of the available reports, sMer was significantly lower in comparison with healthy controls, with no correlation observed between sMer and disease activity scores; conversely, a different study reported increased levels of circulating sMer in RA, however, reiterating the absence of significant correlations with clinical parameters [84].

Lower levels of Gas6, ProS1, and sAxl in RA have also been documented [42, 43]. Gas6 and sAxl, both significantly decreased in patients compared to healthy controls, positively correlated between them; Gas6 also negatively correlated with the presence of erosions and positively with disease activity scores [42]. Because in RA several disease processes occur at the joint site, the discovery that sAxl is one of the most abundant proteins detected in synovial fluid of RA patients suggests that dysregulation of Axl synovial expression may be a pathogenic pathway worth to be explored in future studies [85].

## 6. Conclusion

RA is a chronic inflammatory autoimmune disease affecting joints. Impairment of homeostatic regulators of inflammation likely contributes to the development of persistent inflammatory infiltration of the diseased synovium. Because the defective functionality of TKRs Tyro3, Axl, and Mer (TAM) results in the abnormal activation of the immune system, it has been postulated that these receptors may be implicated in the development of autoimmune diseases including RA.

A protective role for Axl and Mer is supported by finding that induced arthritis is significantly more severe in mice lacking these two receptors. Moreover, Axl likely contributes to physically protecting the joint as it has been found expressed by a special subset of CX3CR1+ lining macrophages originating from synovial precursors and able to form a tight function-mediated barrier. Interestingly, RA-derived DCs have defective Axl expression secondary to the upregulation of its inhibitory micro-RNA miR-34a. Mer, which is typically expressed by anti-inflammatory M2c-polarised macrophages, is upregulated in noninflammatory arthritis like osteoarthritis and RA in remission. Plausibly, Mer plays a crucial role in the synovium by enhancing IL-10, inhibiting proinflammatory cytokines production, and preventing the accumulation of apoptotic cells. In contrast with these results, data about the role of Tyro3 in arthritis showed that its activation is detrimental for the joints as it mediates synovial hypertrophy and increases the erosive burden. Overall, however, the exogenous administration of TAM ligands seems to ameliorate the disease in experimental models of arthritis. Finally, there is growing attention to the quantification of soluble circulating TAM receptors/ligands and its relationship with clinical phenotypes and disease progression.

**Figure 1:**
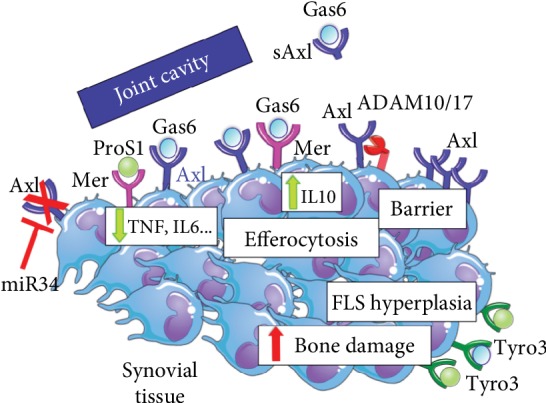
Model of TAM receptors and ligands' effects in synovial tissue. Axl and Mer, once activated by their cognate ligands, exert a protective role within the joint by reducing the production of proinflammatory cytokines, such as TNF and IL-6, and triggering the phagocytosis of apoptotic cells. Axl, specifically, also contributes to form a barrier on the synovial lining while Mer further enhances the anti-inflammatory response by upregulating IL-10. Axl is negatively regulated by miR-34a, which is constitutively activated in RA DCs, and can be cleaved and released as soluble (s) Axl in the joint space by proteinases like ADAM10/17. In contrast, Tyro3 may foster synovial hypertrophy of fibroblast-like-synoviocytes (FLS) and increase bone loss.

In conclusion, available evidence suggests that Axl, Mer, and Tyro3 might play an important and multifaceted role in RA (Figure 1), and further studies on this topic are called to clarify TAMs' role and therapeutic potential.
